# A Case of Amiodarone Pulmonary Toxicity with Short-term Amiodarone Use

**DOI:** 10.7759/cureus.7680

**Published:** 2020-04-15

**Authors:** Abigail McDonald

**Affiliations:** 1 Internal Medicine, HCA Healthcare/USF Morsani College of Medicine GME Programs, Northside Hospital, St. Petersburg, USA

**Keywords:** amiodarone pulmonary toxicity

## Abstract

This is the case of a 92-year-old female who was hospitalized one month prior to admission for symptomatic paroxysmal atrial fibrillation, requiring intravenous amiodarone. Following her previous admission, she was placed on one month of amiodarone 200 mg twice daily, with a one week transition to 200 mg daily. The patient subsequently developed progressive shortness of breath and dry cough over a period of several weeks. Initial imaging showed diffuse bilateral coarse patchy interstitial infiltration on chest X-ray and bibasilar pleural effusions and scattered bilateral opacities on CTA chest. In an elderly patient presenting with dyspnea and dry cough, as well as interstitial opacities on imaging, amiodarone pulmonary toxicity should be considered despite short-term low-dose use.

## Introduction

Amiodarone is a frequently used drug for the management of arrhythmia. It is associated with various pulmonary adverse effects, including but not limited to interstitial fibrosis, hypersensitivity pneumonitis, acute respiratory distress syndrome, and alveolitis. Clinical presentations include gradually worsening dyspnea, dry cough, and pleuritic chest pain [1]. They may also include fevers, weight loss, and hemoptysis [2]. Adverse pulmonary effects may be observed as rapidly as following the first few days of amiodarone use to chronically and insidiously over years [3]. One recent case report of an elderly woman prescribed 400 mg three times daily over two weeks and 200 mg daily thereafter was readmitted within three weeks with symptoms of toxicity [4]. Another recent case report of a 68-year-old female status-post triple vessel coronary artery bypass grafting complicated by atrial fibrillation who received bolus intravenous amiodarone and was discharged on 400 mg PO amiodarone returned to the hospital after 10 days with symptoms of toxicity [5]. Adverse effects may be seen at doses as low as 200 mg daily [6]. Risk factors are not well-defined but may include increasing age, underlying pulmonary disease, cumulative doses, and doses greater than 400 mg [2].

## Case presentation

A 92-year-old non-smoker female with a past medical history of paroxysmal atrial fibrillation on apixaban and metoprolol, tachycardia-bradycardia syndrome with a St. Jude dual-chamber permanent pacemaker, insomnia, hyperlipidemia, hypertension, gastroesophageal reflux disease, and osteoarthritis presented for the evaluation of progressively worsening shortness of breath for the last two to three weeks with acute worsening on the night of admission after being out dancing. The patient had just recently been hospitalized one month and a week prior for symptomatic paroxysmal atrial fibrillation, requiring intravenous (IV) amiodarone and discharged on a regimen of amiodarone 200 mg twice daily for one month, then 200 mg once daily for the last week. The patient reported that even after taking a shower, she feels winded. She additionally reported progressively worsening dry cough and wheezing over the last two to three weeks. The patient denied hemoptysis, productive cough, palpitations, chest pain, orthopnea, lower extremity edema, fevers, chills, weakness, dizziness, and recent illness. The patient additionally reported that since starting amiodarone, she developed blurred vision and tremors that were progressively worsening. The patient reported undergoing recent outpatient pulmonary function testing, which was unremarkable.

**Figure 1:**
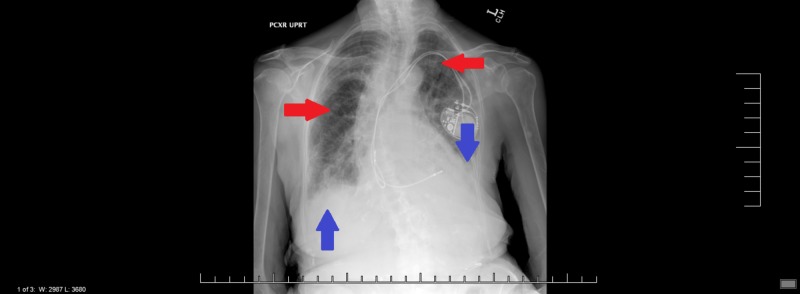
Chest X-ray Diffuse bilateral coarse patchy interstitial infiltrates and pleural effusions Red arrows - interstitial infiltrate, blue arrows - pleural effusions

In the emergency department, the patient’s initial oxygen saturation was 85% and she was mildly tachypneic. The patient was started on a non-rebreather mask, then weaned to a 2L nasal cannula with improvement to oxygen saturation of 96%. The initial chest X-ray showed diffuse bilateral coarse patchy interstitial infiltrates and pleural effusions (Figure 1).

**Figure 2:**
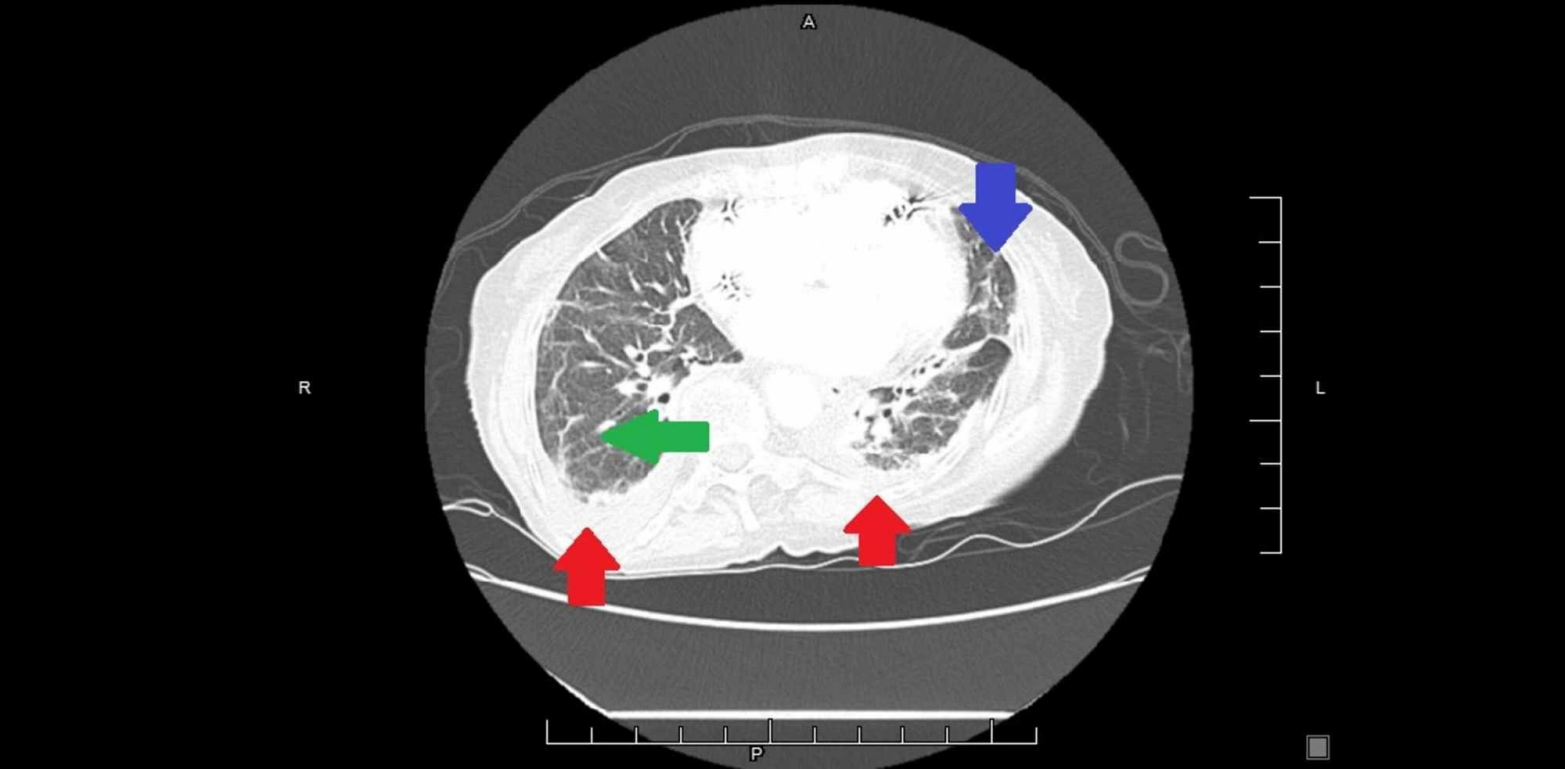
CT angiography of the chest Red arrow - pleural effusion, green arrow - opacity, blue arrow - honeycombing

In the emergency department, D-dimer was found to be elevated and the ensuing bilateral lower extremity venous duplex scan was negative for lower extremity deep vein thrombosis. Computerized tomography angiography of the chest showed no evidence for pulmonary embolism, though bibasilar pleural effusions were noted in addition to scattered bilateral opacities with possible early signs of honeycombing (Figure 2).

**Figure 3:**
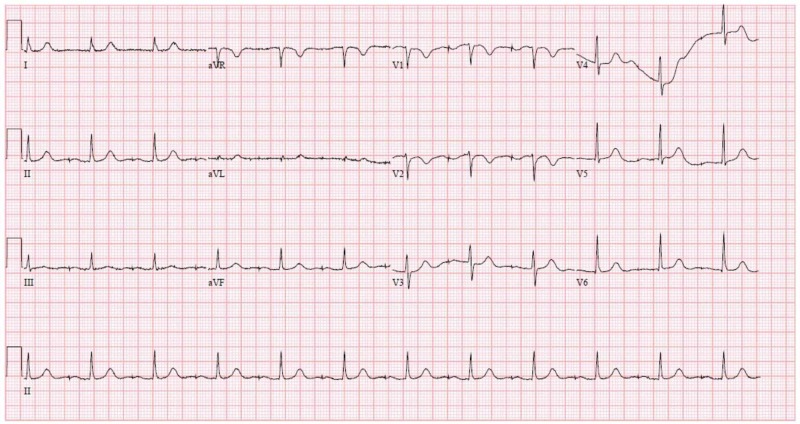
ECG - atrial-paced rhythm, no acute ischemic changes ECG: electrocardiogram

Upon admission, the physical exam was remarkable for bilateral crackles with a Velcro-like quality from the bases to the mid-lung. No wheezing was appreciated. The cardiac exam was unremarkable - the patient had regular rate and rhythm, no murmurs were auscultated, no pitting edema, jugular vein distention (JVD), or carotid bruits noted. The patient was afebrile and appeared to be in no acute distress. The thyroid was not enlarged with no palpable irregularities. The ophthalmic exam was unremarkable. The skin color was noted to be normal. Initial labs showed no leukocytosis and arterial blood gas was consistent with a primary respiratory alkalosis. Troponin was negative, NT-pro-B-type natriuretic peptide was elevated, and procalcitonin was negative. The electrocardiogram (ECG) demonstrated no acute ischemic changes or arrhythmias - heart rate was within normal limits, PR and QT intervals were not elongated, and QRS was narrow (Figure 3).

The echocardiogram showed normal systolic function and the ejection fraction was 60%-65%.

The patient’s amiodarone was stopped and she was given supportive treatment and started on prednisone given concern for possible amiodarone toxicity. As atypical pneumonia could not be wholly ruled out, the patient was started on levofloxacin. The patient subsequently developed urticaria and was switched to doxycycline. Legionella antigen was negative, Mycoplasma pneumonia immunoglobulin M (IgM) was nonreactive, and urine Streptococcus pneumoniae antigen was negative.

The six-minute walk test revealed 87% oxygen saturation on room air at rest and 85% with activity, with improvement to 94% at rest and with activity on a 2L nasal cannula. The patient reported ongoing dyspnea on exertion, though improvement was found as the patient was weaned to a 1L nasal cannula with good saturation. A follow-up chest X-ray prior to discharge continued to show coarse interstitial opacities.

## Discussion

In an early, single-center study of 80 patients being treated with amiodarone for refractory arrhythmias, 5% developed the clinical and radiologic features of interstitial pneumonitis, having no prior history of lung disease. Of these patients with interstitial pneumonitis, 75% were receiving a maintenance dose of 400 mg daily over five to 36 months [1]. One study of 573 patients in a single-center study found the incidence of amiodarone pulmonary toxicity to be 5.8%, occurring between six days and 60 months of treatment, the highest incidence occurring within the first 12 months. Patients older than 62 years developed toxicity more frequently. Patients who started therapy younger than the age of 40 did not develop toxicity in this study. Other risk factors included lower pre-amiodarone treatment diffusing capacity for carbon monoxide (DLCO) and higher daily average maintenance dose. The development of toxicity was not predicted with factors, including gender, arrhythmia, lung volume abnormalities, or underlying heart disease [7]. In this case, the patient was at risk given her age and incidence of possible amiodarone toxicity occurring within her first 12 months of therapy.

Several possible mechanisms are proposed regarding amiodarone pulmonary toxicity. One such mechanism is via inflammatory or immune effector cells. Another mechanism is direct toxicity resulting in lung parenchymal cell injury [8]. One study indicated that in vitro amiodarone could induce bovine pulmonary artery endothelial cells to form lamellar cytoplasmic inclusions, occurring as soon as four hours after incubation with amiodarone with as little as 1 microgram/ml [9]. Another study found pulmonary fibrosis to be mediated by the dose-dependent induction of apoptosis and necrosis in vitro (rat alveolar epithelial cells and human-derived alveolar epithelial cells). Cytotoxicity was significant at below-therapeutic concentrations of amiodarone and severe at concentrates known to accumulate in human lung tissue. Interestingly, apoptosis was inhibited by captopril (angiotensin-converting enzyme (ACE) inhibitor) and saralasin (partial angiotensin II receptor agonist) [10].

At present, there is no gold standard for the diagnosis of amiodarone-induced pulmonary toxicity. Other causes must be ruled out, including other pulmonary conditions such as chronic obstructive pulmonary disease (COPD) or pulmonary infarct, pulmonary embolism, infectious, cardiac, and malignant (carcinoma, lymphoma). Pulmonary function testing demonstrates restrictive defects with reduced diffusion capacity [1]. One study found a median TLC of 78%, with diffusion defects in 45% of participants. TLC and DLCO can improve but not significantly in the long term. VC, however, can improve significantly between one and 36 months. A small number of patients may have persistent restrictive syndromes. A chest X-ray can show bilateral opacifications. A resolution of findings can occur over two to eight weeks. High-resolution CT can reveal diffuse bilateral ground-glass opacification, alveolar consolidation, intra-alveolar reticulations, and pleural effusions [11]. In this case, patient history, exam, and workup, including ECG, troponin, and echocardiogram, make cardiac pathology less likely. Recent outpatient pulmonary function testing was negative, though it may be of consideration to obtain new testing given that the patient has significant imaging findings and clinical complaints. Extensive infectious workup is being pursued and prophylactic antibiotics are prudent as atypical pneumonia cannot be completely ruled out at this time. Clinical presentation, in addition to imaging findings, is less likely to support malignancy.

At present, there are no controlled trials to demonstrate the efficacy of any given treatment. Amiodarone cessation can result in the improvement of clinical presentation over two to eight weeks [1]. Imaging and pulmonary function do not tend to improve as quickly as clinical presentation. Discontinuation alone may be sufficient if the disease is limited. In patients who show a substantial involvement on imaging studies, or if patients are hypoxic, systemic corticosteroids may be utilized [12]. Treatment with systemic corticosteroids was found in one study to induce relapse when treatment lasted for less than three months but not longer than six months, probably attributable to the 40-70 day half-life elimination of amiodarone and storage in the lung parenchyma. Positive effects were noted on alveolar opacifications [11]. An initial dose of 0.75-1 mg/kg of prednisolone should be maintained until there is a definite clinical and radiographic response, as recurrence can be more severe than the initial episode. If no improvement is found after one to two months of systemic corticosteroids, alternative diagnoses should be considered. Unfortunately, amiodarone-induced fibrosis can be irreversible with poor response to corticosteroids. Acute respiratory distress syndrome (ARDS) in the setting of amiodarone toxicity is rapidly progressive, often requiring mechanical ventilation, and is also found to have a poor response to systemic corticosteroid therapy [12]. Another study investigating the effects of captopril and the angiotensin receptor antagonist losartan found that alveolar wall collagen accumulation was significantly reduced by the administration of captopril (100%) or losartan (70%), but these agents had no effect on the accumulation of alveolar macrophages stimulated by amiodarone, suggesting an effect on fibrosis and not alveolitis [13]. A following eight-year retrospective single-center analysis of 1000 patients on amiodarone was conducted, finding amiodarone pulmonary toxicity in 2.2% of that population, with the occurrence of toxicity in 1% of patients already on an ACE-I or angiotensin II receptor blocker (ARB) and in 3.9% of patients not taking an ACE-I or an ARB, suggesting a possible protective effect of ACE-Is/ARBs against amiodarone pulmonary toxicity [14]. A case study conducted on a patient with severe toxicity and rapid onset respiratory failure refractory to corticosteroid therapy on mechanical ventilation was started on polymyxin B-immobilized fiber column direct hemoperfusion (PMX-DHP), a device that reduces blood endotoxin levels generally used in cases of sepsis. Clinical improvement of respiratory failure was observed with a reduction in the serum levels of amiodarone and its metabolite. The patient did well without recurrence [15]. If no infectious source is found, this patient may benefit from continued systemic corticosteroid treatment for another one to two months with re-evaluation for clinical and imaging improvement. As the patient has failed her six-minute walk test, she will need to go home with oxygen therapy. Given the patient’s history of hypertension, the initiation of an ACE inhibitor or ARB may be considered for protection against worsening fibrosis. The patient will need to remain off of amiodarone therapy, and alternative medications to protect against arrhythmia may be pursued.

## Conclusions

Although the incidence of amiodarone pulmonary toxicity is relatively low, especially with dosing less than 400 mg daily, its possibility in an elderly patient should be considered. Adverse effects have been noted with amiodarone at doses as low as 200 mg daily and as soon as within the first few days of amiodarone use. Additionally, cytotoxicity has been noted at below-therapeutic concentrations. Future studies should be pursued to determine the efficacy of treatments for amiodarone pulmonary toxicity, standardizing diagnosis, as well as better characterizing amiodarone pulmonary toxicity in the setting of short-term therapy.
